# SauDial: The Saudi Arabic dialects game localization dataset

**DOI:** 10.1016/j.dib.2025.111906

**Published:** 2025-07-20

**Authors:** Naif Alanazi, Mohammed Al-Batineh, Hussein Abu-Rayyash

**Affiliations:** a Department of English, Prince Sattam bin Abdulaziz University, Al-Kharj, Saudi Arabia; b Department of Languages and Literature, United Arab Emirates University, United Arab Emirates; c Department of Modern & Classical Language Studies, Kent State University, Kent, USA

**Keywords:** Game localization, Cultural adaptation, Arabic gaming, Dialect corpus, Large language models

## Abstract

Content creation and localization for video games demand substantial effort from script writers and localization teams. Consequently, we present SauDial, the Saudi Arabic Dialects Game Localization Parallel Dataset, a collection of Saudi dialectal expressions tailored for localization-related tasks. The corpus features samples from four Saudi dialects, namely Najdi, Hijazi, Janoubi, and Eastern. The dataset was first produced through an AI‑driven process informed by cultural knowledge, linguistic expertise, and game‑specific context, then manually cleaned, refined, and revised to ensure dialectal accuracy, tonal appropriateness, and cultural and semantic fidelity. Each entry contains an English source line, a Modern Standard Arabic (MSA) translation, and a dialectal counterpart together with context clues, age ratings, and linguistic notes. The dataset spans a broad array of scenarios relevant to multiple game genres and tonal indicators, and it aligns with the General Authority of Media Regulation (GCAM) official rating system. In addition, it opens avenues for research in Translation, Cultural, Localization, and Game Studies, while in educational settings it can support translation and localization courses and serve as a translation memory that aids professional translators and localizers. To the best of our knowledge, SauDial is the first dataset of its kind in game localization and offers a foundation that can strengthen the authenticity and cultural resonance of games localized for the Saudi market.

Specifications Table

**Table:** 

Subject	Computer Sciences, Social Sciences
Specific subject area	Large-language Models (LLMs), natural language processing (NLP), machine translation (MT), game Localization, translation studies, cross-linguistic analysis
Type of data	Textual data, translated and aligned
Data collection	The dataset was generated through Application Programming Interface (API) calls to OpenAI's GPT-4o model. A Python script sends prompts that combine the designated Saudi dialect with a scenario, game genre, tone, and age rating, then retrieves the output: the English line, its MSA translation, the dialectal rendering, contextual explanations, and linguistic notes. The script labels each entry with a localization-difficulty level and target-audience category, compiles the records, and exports them to an Excel spreadsheet. Data quality was safeguarded through error handling, content-uniqueness checks, and an option for user interruption.
Data source location	The primary data sources comprise OpenAI GPT-4o model responses and pre-compiled dialect-specific resources integrated within the python script.
Data accessibility	Repository name: Mendeley Data Data identification number: 10.17632/mzdwkb2t6d.1 Direct URL to data: https://data.mendeley.com/datasets/mzdwkb2t6d/1
Related research article	*‘*none’

## 1 Value of the Data

• Assist in developing narratives for Arabic for Generative Non-Player Characters (NPCs) in Video Games [1] and providing specialized dialect-specific training data crucial for AI-driven video game localization into Saudi Arabic dialects.
• Provide specialized dialect-specific training data crucial for AI-driven video game localization into Saudi Arabic dialects.
• Leverage in translation studies to compare translation strategies across different Saudi dialects and to examine how figurative language and cultural references move between Saudi regional varieties.
• Facilitate the process of game glocalization through empowerment of local identity [2], moving away from locale-based localization to a more micro level localization at the level of dialect within a certain locale.
• Investigate how implementing various Saudi dialects influences player engagement and immersion, generating insights into regional preferences that guide game developers and localizers in selecting effective localization strategies.
• Support professional and educational applications by serving as a practical and authentic teaching tool for training students in translation and localization while functioning as a ready-to-use translation memory (TM) for professional translators and localization teams.

## 2 Background

The SauDial dataset is conceived to fill a gap in Arabic language resources for game localization, especially Saudi dialects. Its creation answers the surge in the Saudi game market, a growth spurred by initiatives from Savvy Games Group (SGG), a subsidiary of Saudi Arabia’s Public Investment Fund and part of Vision 2030. The Saudi government plans to invest $38 billion in the video‑game sector by 2030 [3], a figure that underscores the industry’s strategic importance and expansion potential. In this landscape, SauDial’s linguistic representation can boost player engagement and cultural resonance, thereby advancing Saudi Arabia’s economic and cultural goals. The dataset also acts as a launchpad for the production of authentic content, deeper localization into regional dialects, and a more culturally and linguistically engaging gaming experience.

The creation of this dataset was informed by sociolinguistic theories on dialect variation, such as Speech Community Theory [4], Communication Accommodation Theory [5], and the principles of dynamic equivalence in translation studies [6]. It goes beyond simple lexical differences in Saudi dialects and takes into consideration more pragmatic and cultural aspects that are necessary in game localization [7].

The methodology leveraged an advanced reasoning LLM (GPT-4o) to generate initial content that was then refined by linguistic expertise, similar to the approach used by [8]. This methodological approach allowed the rapid creation of a diversified corpus while maintaining cultural and dialectical accuracy through human oversight.

The employment of generative AI in dataset creation was prompted by the shortage of natural dialectal content for gaming and the necessity for systematic handling of linguistic parameters that natural corpus collection approaches could not handle efficiently. Hand creation of the content or corpus compilation would require extensive fieldwork in different Saudi areas and considerable time to achieve equivalent coverage of gaming scenarios, emotional shades, and dialect types. The LLM-powered approach facilitates rapid generation of diversified, contextually appropriate dialogue subject to systematic control over parameters such as dialect type, cultural setting, and age appropriateness to build a balanced dataset through human-AI collaboration leveraging automated scalability with professional linguistic authenticity guarantee.

SauDial addresses the scarcity of dialect-specific content in Arabic natural language processing (NLP) and video game localization. It will be a valuable tool for localizers, researchers, and linguists seeking to develop better language technologies, enhance gaming experiences, and support sociolinguistic explorations of Saudi dialects in contemporary digital ecologies.

## 3 Data Description

SauDial provides localized game dialogue across various Saudi dialects. It contains columns that describe the scenarios, game genre, tone, age rating, and translations in both MSA and specific dialects. This dataset is structured to aid game localizers in localizing games for Saudi audiences by providing cultural and dialect-specific translations.

### 3.1 Dataset structure and columns

1. **Dialect**: Indicates the Saudi dialect in focus (e.g., Najdi, Hijazi, Eastern, Southern).
2. **Scenario**: Provides the specific gaming context (e.g., ``Desert Racing,'' ``Jeddah Tower Escape Room'').
3. **Game Genre**: Identifies the genre of the game (e.g., Action, Puzzle, Simulation). Fig. 1 (below) visualizes the frequency of different game genres in the dataset.

**Fig. 1:**
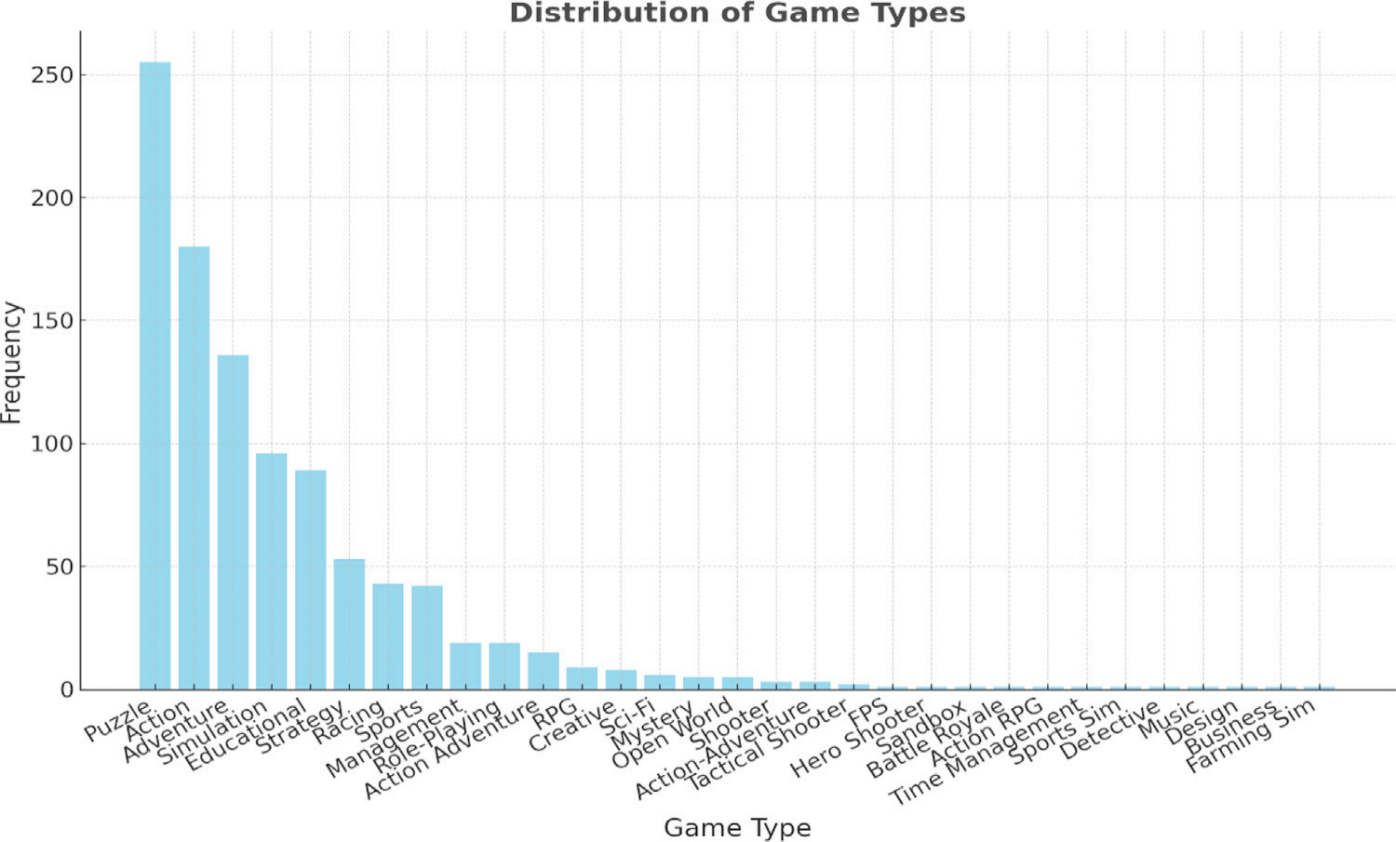
Distribution of game types.
  Fig. 1 visualizes the frequency of different game genres in the dataset, highlighting the variety of game types covered.
4. **Tone:** The emotional tone of the dialogue defines how a character feels and how that feeling is conveyed to players (e.g., Excited, Mysterious, Reflective). Therefore, these tone categories are essential for achieving what experts in game localization refer to as emotional localization, a concept sometimes described as “emotion engineering” or kansei [9]. This involves reproducing the same emotional experience across languages and cultures so that players everywhere feel the same impact intended by the game’s creators [10].

To achieve this, we developed distinct linguistic and emotional characteristics for each tone. For example:

• *Serious* employs formal language with minimal use of colloquialisms.
• *Humorous* integrates jokes, puns, and playful expressions.
• *Whimsical* features imaginative, light-hearted phrasing.
• *Mysterious* leans into ambiguity and suspense.
• *Inspirational* delivers messages of motivation and encouragement.
• *Dark humor* combines wit with macabre or morbid elements.
• *Contemplative* presents introspective, thought-provoking lines.
• *Fearful* communicates anxiety, tension, or dread.
• *Excited* is energetic and enthusiastic in tone.
• *Reflective* focuses on reminiscence and emotional retrospection.

As Díaz Montón points out, ``Dialogue lines […] are one of the most creative parts of game content,'' wherein translators can tease out ``the nuances of the game characters'' [9]. Applying these definitions of tone to both content creation and validation not only allowed for coherence, but also empowered localizers to actually preserve each character's voice and emotional resonance through the four primary Saudi dialects.

**5. Age Rating:** Specifies the recommended age group for each dialogue (+3, +7, 12+, +16, 18+).

**6. English Text:** The original game dialogue in English.

**7. MSA Translation:** Provides the translation of the English text into MSA.

**8. Dialect Translation:** Offers localization specific to the dialect mentioned in the first column, including unique dialect-specific vocabulary and expressions.

**9. Context and Rating:** Describes the cultural and contextual background of the dialogue and its suitability for various age groups.

**10. Dialect Notes:** Includes linguistic notes about dialect-specific usage, terminology, and idiomatic expressions used in the localization.

**Fig. 2:**
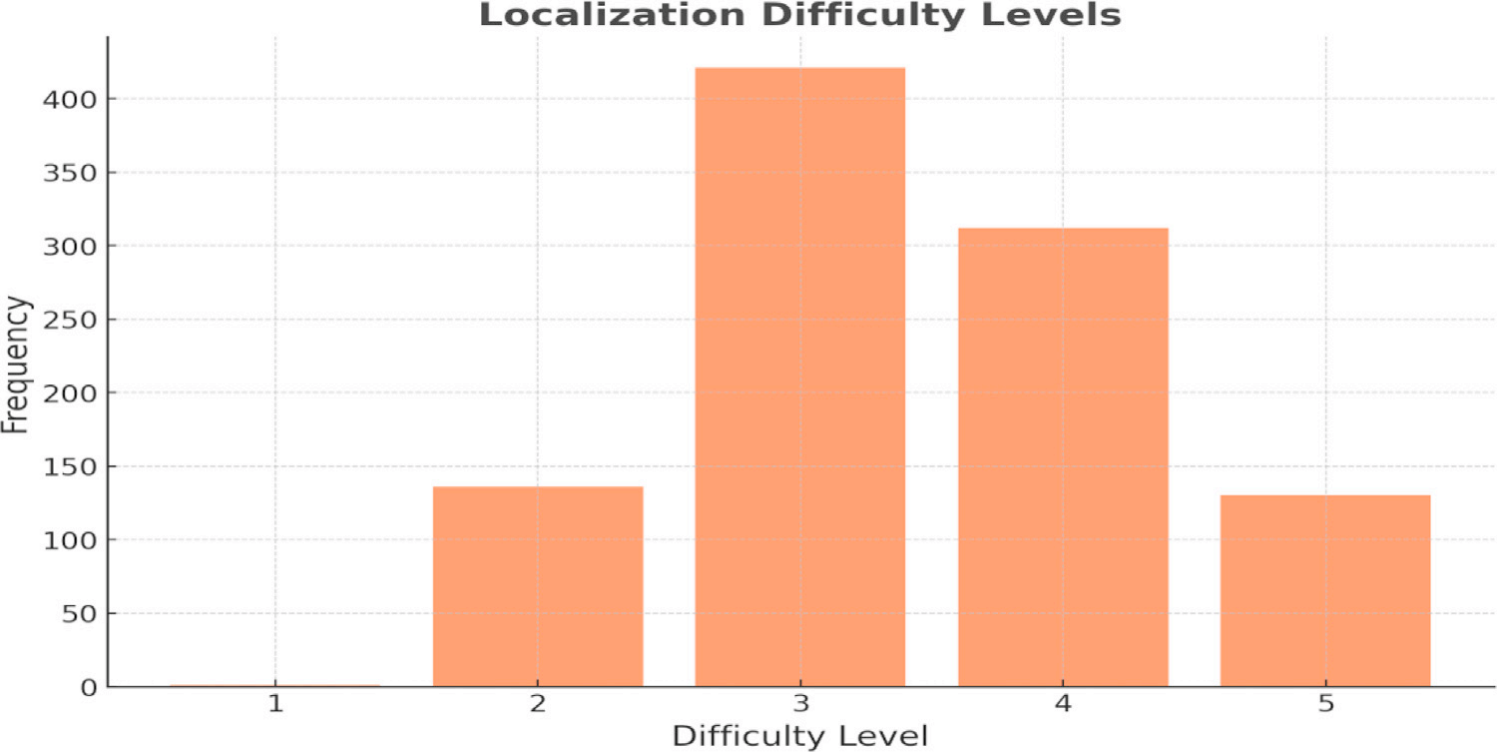
Localization difficulty levels.

**11. Localization Difficulty:** The rating proposed here reflects the complexity involved in accurately translating the content while preserving cultural subtleties and dialectal authenticity. This complexity is also shaped by the broader context of the language industry, where localization workflows remain largely non-standardized [11]. Furthermore, video game localization, as a distinct subfield, involves adapting a wide range of assets, including dialogue, cultural content, legal documents, and marketing materials, which underscores the multifaceted nature of this task [9]. In response to this lack of standardization, the difficulty was assessed on a 5-point scale (1–5) by the authors, who are localizers with over five years of professional experience. The authors were actively involved in every stage of the review process, including annotation validation, dialect identification, and tone confirmation. The evaluation followed a well-defined set of criteria: 1) lexical complexity (availability of dialect-specific equivalents), 2) cultural specificity (presence of culture-bound elements requiring adaptation), 3) pragmatic functions (speech acts that vary significantly across dialects), and 4) technical terminology (game-specific jargon). Higher ratings indicate content requiring more sophisticated localization strategies and deeper cultural knowledge. Fig. 2 (below) illustrates the levels of difficulty associated with localizing the game dialogues. This chart provides insights into the localization challenges across different contexts.

Fig. 2 displays the difficulty of localizing dialogues into various Saudi dialects.

**12. In-Game Context:** Explains the role or activity the player is involved in during the game, providing insight into how the dialogue is used in the gameplay.

SauDial is organized for easy reference and utility in game localization, ensuring that localizers can create culturally appropriate and engaging content for Saudi gamers*.*

## 4 Experimental Design, Materials and Methods

The SauDial dataset was created using a novel approach that combines advanced language model capabilities with human linguistic and cultural expertise. This section outlines the methodology used to generate, refine, and validate the dataset.

### 4.1 Data generation framework

The core of the data generation process was implemented using a python script, leveraging the OpenAI API to interact with the GPT-4o model. The script was developed to systematically generate diverse game localization content across various Saudi Arabic dialects.

Key components of the script include:

**a) OpenAI API Integration:** The script uses the OpenAI Python library to make API calls to the GPT-4o model. OpenAI's GPT-4o model was selected among other generative AI tools (Claude AI, DeepSeek AI, Meta AI, Gemini AI, etc.) for its superior performance in maintaining Arabic dialectal stability, reducing cross-regional contamination, and providing stable API infrastructure for mass-scale dataset generation. Early comparative testing revealed that GPT-4o generated the most accurate production of region-specific linguistic features due to large multilingual training data, but with technical stability, consistent response formatting, and sustainable token usage rates to produce the entire 1000-entry dataset with complex prompt engineering scenarios with multiple contextual variables.

**b) Dialect Resources:** A dictionary framework was created to store dialect-specific resources like idioms, cultural allusions, pronunciations, and common words in every one of the four major Saudi dialects: Najdi, Hijazi, Janoubi, and Eastern. The structure of the framework combined traditional lexicographic effort and modern computational tools. [12] was the main lexical reference for Najdi varieties, [13] for Eastern dialects, and [14] for cross-regional contrast. These were supplemented with contemporary corpus data from the MADAR project [15], which offered parallel translations for Saudi cities, and the Najdi Arabic Corpus [16], providing 275,134 tagged tokens to be computationally checked.

**c) Dynamic Prompt Generation:** The script generates prompts for the LLM model, incorporating specific dialect features, scenario types, game genres, tones, and age ratings.

**d) Content Generation and Validation:** The ‘generate content’ function makes API calls to generate localization content and parses the response into a structured format.

**e) Data Collection and Storage:** The ‘collect data’ function generates content for various combinations of dialects, scenarios, game types, tones, and age ratings. Generated content is stored in a list of dictionaries, which is later converted to a pandas DataFrame for easy manipulation and export.

### 4.2 Experimental parameters

The dataset was generated using the following parameters:

**a) Dialects:** Najdi, Hijazi, Janoubi, Eastern

**b) Scenario Types:** 26 different scenarios, including 'epic battle', 'marketplace haggling', 'family gathering', 'desert adventure', etc.

**c) Game Genres:** RPG, Adventure, Platformer, Action, Simulation, Strategy, Puzzle, Educational

**d) Tones:** Serious, Humorous, Whimsical, Mysterious, Inspirational, Dark humor, Contemplative, Fearful, Excited, Reflective

**e) Age Ratings:** For the age rating parameters, three rating systems were initially considered: the Pan European Game Information (PEGI), the Entertainment Software Rating Board (ESRB), and Saudi Arabia's official age rating system (GAMR) for video games. Given the specific focus of this dataset on Saudi dialects, the Saudi age rating system was adopted for assessing and ranking the content generated for the game scenarios.

### 4.3 Linguistic resources and validation

To ensure the dialectal authenticity of the SauDial dataset, we drew on a wide range of trusted linguistic sources, including traditional dictionaries, modern language corpora, and advanced computational tools.

Lexical documentation began with key regional dialectology sources. Najdi dialect features were drawn from Ingham’s comprehensive exploration *Najdi Arabic: Central Arabian* [12], based on 15 years of immersive fieldwork. For Eastern dialects, the work *Dialect, Culture and Society in Eastern Arabia* provided detailed glossaries and rich ethnographic insights [13]. A broader look at cross-regional variations was offered by *Saudi Arabian Dialects* [14]. More recent usage patterns were examined using data from the MADAR Project corpus, which includes parallel translations from 25 Arabic city dialects, including major Saudi cities [15]. Furthermore, computational checks made use of a range of modern tools. The Najdi Arabic Corpus was used to validate part-of-speech patterns and word frequency [16].

Additional linguistic insights were drawn from academic studies and institutional sources. [17] work on regional dialect leveling informed our understanding of Najdi Arabic phonology, while the scarce documentation on southern dialects was supported by [18] analysis of the Abha dialect and [19] sociolinguistic research on Jazani Arabic. To enrich the lexical scope, we incorporated specialized dictionaries from the King Salman Global Academy for Arabic Language and consulted the Saudi Data and AI Authority’s glossary for technical terminology. For underrepresented dialects, particularly Janoubi varieties, we supplemented formal sources with community-based online materials and verified authenticity through post-generation review.

### 4.4 Content generation process

The content generation process follows these steps:

**a)** For each combination of dialect, scenario, game type, tone, and age rating, the script generates a dynamic prompt.

**b)** This prompt is sent to the GPT-4o model via the OpenAI API.

**c)** The model generates content including English text, MSA translation, dialect translation, context and rating explanation, and dialect notes.

**d)** The generated content is parsed and stored in a structured format.

**e)** Additional metadata such as word counts, localization difficulty, target audience, and in-game context are added to each entry.

### 4.5 Data validation and refinement

To ensure the quality, authenticity, and originality of the generated content across the whole 1000-entry dataset, a multi-layered validation and refinement process was used, intermixing automated quality control procedures with systematic human expert assessment.

**a) Technical Quality Assurance**

The automated validation pipeline had several mechanisms in place to ensure data integrity in generating all 1000 entries. First, a deduplication system based on hashes was employed to prevent storing semantically equivalent content such that every entry constitutes a representation of another localization scenario with distinct linguistic and contextual characteristics. Second, comprehensive error handling and exponential backoff retry logic were implemented in the API communication protocols to manage likely failures, timeouts, and rate limiting issues that would impact quality in content generation. Third, automatic quantitative metrics like English source text word counts, MSA translation word counts, and dialect translation word counts were calculated for each entry, which provide statistical data on translation expansion and contraction patterns between linguistic varieties and for quality evaluation.

**b) Human Expert Review Process**

Other than technical automation verification, the entries produced were manually checked in their entirety by a sole expert refiner from the authorship team, a qualified localizer with over five years of exclusive experience in Arabic dialect translation and game localization. This one-reviewer approach ensured the same assessment criteria and guaranteed identical quality standards across the entire dataset, eliminating potential inter-rater variability while employing in-depth domain knowledge of Saudi dialect varieties.

The fine-tuning process addressed three categories of errors commonly produced by large language models during the generation of dialectal output. Cross-regional dialect pollution was addressed and eliminated first, whereby AI models incorrectly mixed linguistic features of Saudi dialects of different regions, producing artificial hybrid varieties. Generic dialectal output without regional feature specificity essential to true localization was replaced with relevant region-specific alternatives. Third, an inappropriate linguistic register that was not conveying the intended emotional tone and indicated cultural context provided in the generation parameters was calibrated to achieve the target communicative objective.

Table 1 illustrates a common instance of this human-made adjustment procedure, and the unique types of repairs made to ensure dialectal authenticity and regional specificity across the dataset are highlighted.

**Table 1:** Hijazi dialect - modern city life (humorous tone).

As illustrated in Table 1, the original LLM-generated content tended to reflect cross-regional blending of dialects, for example, inappropriate use of Gulf Arabic intensifier  as opposed to the accurate Hijazi form  and generic words that failed to capture region-specific linguistic practice and cultural patterns. The processed version invariably uses authentic dialect-specific words, culturally appropriate terminologies, and proper tonal consistency that best captures the sociolinguistic fact of each Saudi dialect variety.

**c) Quality Standards and Calibration**

The expert refining process adhered to standard evaluation criteria to ensure that each of the 1000 entries still had four critical dimensions of quality: (a) lexical authenticity by using documented lexemes specific to dialect from the huge library of linguistic materials outlined above, (b) cultural accuracy in portraying regional traditions, social environments, and contemporary usage patterns, (c) appropriate pragmatic functions as pertaining to the intended use communicative function and game context situation, and (d) technical jargon for the suggested game genre and age rating.

In addition, refining was a process that involved difficulty rating calibration of localization based on the complexities observed through manual inspection patterns. This was to ensure that the 1–5 scale corresponded with actual real-world difficulties faced by professional localizers when handling similar content in game localization scenarios, providing valuable metadata for future applications of the dataset.

This human-in-the-loop process was essential to creating a high-quality dataset that serves to accurately represent the linguistic and cultural richness of Saudi dialectal varieties without compromising on consistency, accuracy, and professional applicability across all 1000 entries. The resultant dataset offers a sound foundation for academic studies as well as real-life applications in Arabic game localization.

### 4.6 Data storage and presentation

The complete dataset comprising 1000 generated entries is stored in a structured Excel file designed for optimal accessibility and usability:

1. Each row represents a unique localization entry.
2. Columns include Dialect, Scenario, Game Type, Tone, Age Rating, English Text, MSA Translation, Dialect Translation, Context and Rating, Dialect Notes, Localization Difficulty, and In-Game Context.
3. The Excel file is formatted for readability, with column widths adjusted automatically. Dialect-specific words in the 'Dialect Translation' column are highlighted in red for easy identification.

The Excel file is formatted for readability, with column widths adjusted automatically. Dialect-specific words in the 'Dialect Translation' column are highlighted in red for easy identification.

## Limitations

While the methodology leverages LLM capabilities, there are a few caveats. AI-generated content may not always perfectly capture the nuances of dialect use, and it needs to be edited and fine-tuned by native speakers and subject-matter specialists before use for professional use. Availability of fine-grained lexical resources for the four Saudi dialects varies significantly, with more documentation available for Najdi and Eastern varieties [12,13] compared to Southern (Janoubi) varieties, where we were mostly dependent on limited academic research [18,19]. The disparity of source materials can affect the degree of detail and representativeness of less-documented varieties.

## Ethics Statement

The authors confirm that they have read and follow the ethical requirements for publication in Data in Brief. We hereby state that the current work does not involve human subjects, animal experiments, or any data collected from social media platforms. The SauDial dataset was generated using artificial intelligence models and does not contain any personal or identifiable information. All content was created synthetically for the purpose of game localization research and development, adhering to ethical guidelines for AI-generated content. The age ratings and cultural references used in the dataset are based on publicly available information and do not reflect real individuals or specific events.

## CRediT Author Statement

**Naif Alanazi:** Conceptualization, Resources, Writing - Original Draft, Writing - Review & Editing, Supervision, Project Administration. **Mohammed Al-Batineh:** Writing, Review & Editing, Methodology, Resources. **Hussein Abu-Rayyash:** Conceptualization, Methodology, Software, Data Curation, Writing - Original Draft, Writing - Review & Editing, Visualization.

## Data Availability

Mendeley DataSauDial: The Saudi Arabic Dialects Game Localization Dataset (Original data).
